# Phage Endolysin LysP108 Showed Promising Antibacterial Potential Against Methicillin-resistant *Staphylococcus aureus*

**DOI:** 10.3389/fcimb.2021.668430

**Published:** 2021-04-15

**Authors:** Yifei Lu, Yingran Wang, Jing Wang, Yan Zhao, Qiu Zhong, Gang Li, Zhifeng Fu, Shuguang Lu

**Affiliations:** ^1^ Institute of Burn Research, Southwest Hospital, State Key Lab of Trauma, Burn and Combined Injury, Army Medical University, Chongqing, China; ^2^ Department of Clinical Laboratory Medicine, Southwest Hospital, Army Medical University, Chongqing, China; ^3^ Department of Microbiology, College of Basic Medical Science, Army Medical University, Chongqing, China; ^4^ Department of Clinical Laboratory Medicine, Daping Hospital, Army Medical University, Chongqing, China; ^5^ College of Pharmaceutical Sciences, Southwest University, Chongqing, China

**Keywords:** phage endolysin LysP108, antibacterial activity, drug-resistant bacteria infection treatment, vancomycin, methicillin-resistant *Staphylococcus aureus*

## Abstract

As a potential antibacterial agent, endolysin can directly lyse Gram-positive bacteria from the outside and does not lead to drug resistance. Considering that XN108 is the first reported methicillin-resistant *Staphylococcus aureus* (MRSA) strain in mainland China with a vancomycin MIC that exceeds 8 µg mL^-1^, we conducted a systematic study on its phage-encoded endolysin LysP108. Standard plate counting method revealed that LysP108 could lyse *S. aureus* and *Pseudomonas aeruginosa* with damaged outer membrane, resulting in a significant reduction in the number of live bacteria. Scanning electron microscopy results showed that *S. aureus* cells could be lysed directly from the outside by LysP108. Live/dead bacteria staining results indicated that LysP108 possessed strong bactericidal ability, with an anti-bacterial rate of approximately 90%. Crystal violet staining results implied that LysP108 could also inhibit and destroy bacterial biofilms. *In vivo* animal experiments suggested that the area of subcutaneous abscess of mice infected with MRSA was significantly reduced after the combined injection of LysP108 and vancomycin in comparison with monotherapy. The synergistic antibacterial effects of LysP108 and vancomycin were confirmed. Therefore, the present data strongly support the idea that endolysin LysP108 exhibits promising antibacterial potential to be used as a candidate for the treatment of infections caused by MRSA.

## Introduction

The abuse of antibiotics has led to the frequent emergence of multidrug-resistant superbugs, which make the clinical treatment of infectious diseases difficult (Nathan and Cars, 2014; Blair et al., 2015). Moreover, due to the slow development of new antibiotics, drug-resistant bacteria have become one of the greatest threats to global public health (Woolhouse and Farrar, 2014; Marston et al., 2016). *Staphylococcus aureus* is a common pathogen that causes various human infectious diseases (Suaifan et al., 2017). The emergence of methicillin-resistant *S. aureus* (MRSA) and its rapid development of drug resistance have made the clinical treatment of *S. aureus* infections very challenging (Rodvold and McConeghy, 2014). In recent years, some MRSA strains are reportedly able to resist vancomycin, which has long been considered as the last line of defense against MRSA (Staub and Sieber, 2009; McGuinness et al., 2017). Therefore, the development of new antimicrobial agents for the efficient prevention and treatment of MRSA infections is necessary.

As natural predators of bacteria, bacteriophages (phages) are used in the treatment of drug-resistant bacterial infections (Kutateladze and Adamia, 2010; Díez-Martínez et al., 2015). Besides the phage itself, phage-encoded endolysins can also selectively and quickly kill bacteria, and they showed great potential for application to the treatment of antibacterial infection (Huskins and Goldmann, 2005; Payne, 2008). Endolysin is a cell wall hydrolase that plays an important role in the later stage of phage infection (Gerstmans et al., 2018). It can lyse the cell wall from inside the bacteria and help release progeny phages outside the cell (Rodríguez-Rubio et al., 2013). Compared with phage, endolysin has many advantages, such as non-proliferation, easy-to-target drug delivery, wider host spectrum, and low bacterial resistance (Nelson et al., 2001; Nelson et al., 2012). As a biological macromolecule, its regulatory path is clearer than that of phages.

At present, the development of phage-related preparations that can lyse pathogens has become the focus of research. Since Gram-positive bacteria do not have an outer membrane, endolysin can directly lyse the cell wall from the outside, thereby providing a basis for the use of endolysin alone (Ahmed et al., 2014; Haddad Kashani et al., 2017; Chang, 2020). Considering the synergistic effect between the endolysin and antibiotics, the use of the endolysin in combination with antibiotics can expand the broad spectrum of the endolysin and avoid the development of resistance of target cells (Djurkovic et al., 2005; Schuch et al., 2014; Schmelcher et al., 2015). Some phage endolysin-related preparations are currently in clinical trials or already in the market (Jun et al., 2017; Totté et al., 2017). Therefore, endolysins bring new hope for the treatment of superbugs when antibiotics are ineffective against multidrug-resistant superbugs.

In this study, the endolysin LysP108 was derived from phage P108 of *S. aureus* strain XN108 and obtained by recombinant expression in *Escherichia coli* by molecular cloning. Considering that XN108 is the first MRSA strain in mainland China with vancomycin MIC that exceeds 8 µg mL^-1^ (Zhang et al., 2013; Wang et al., 2020), we conducted a systematic study on its phage-encoded endolysin LysP108. The lysozyme activity of endolysin LysP108 was analyzed by viable count method to determine the optimum conditions of LysP108. Afterward, its *in vitro* bactericidal capacity and its effect on bacterial biofilm were investigated by using live/dead bacteria and crystal violet staining experiments, respectively. To verify the antibacterial effect of LysP108 *in vivo*, a mouse model of subcutaneous MRSA infection abscess was established. Then, LysP108 was used in combination with vancomycin to verify their synergistic effect and assess the possibility of using such a combination as a potential antimicrobial agent. In summary, we have validated the antibacterial effect of LysP108 *in vitro* and *in vivo* and explored its potential in combination with antibiotics, thereby providing a new way for solving the increasingly serious problem of bacterial drug resistance.

## Materials and Methods

### Ethics Statement

All experiments were conducted with the approval of the Laboratory Animal Welfare and Ethics Committee of Third Military Medical University (NO. AMUWEC2020733) and in strict accordance with ethical principles. All participants were informed the purpose of this study and agreed to written consent.

### Materials and Reagents

PCR primers with sequences were synthesized by Genomics institution (China). FastPfu DNA polymerase, *Escherichia coli* strains DH5α and BL21 (DE3) were purchased from TransGen Biotech (China). Quickcut restriction endonucleases NotI and NdeI were both purchased from Takara Bio (Japan). T4 DNA ligase was purchased from BioLab (U.S.A.). IPTG was purchased from Sangon (China). DNA marker, protein marker and live/dead bacteria staining kit were all purchased from Thermo Fisher Scientific (U.S.A.). LB broth and BHI broth were purchased from Oxoid (U.K.). Bicinchoninic acid assay kit was purchased from Beyotime Biotechnology (China). Vancomycin was purchased from Solarbio Life Sciences (China).

### Apparatus

PCR experiments were conducted on a PCR instrument and a gel imager (Bio-Rad Laboratories, U.S.A.). Nickel affinity chromatography was conducted on an AKTA purifier equipped with a HisTrap FF column (GE Healthcare, U.S.A.). Scanning diagram of plate colony was conducted on an automatic colony counter (Shineso, China). Fluorescent micrographic imaging of stained bacteria was conducted on a NI−U fluorescent microscope (Nikon, Japan). OD_570_ value was measured on a SpectraMAX M2e plate reader (Molecular Device, U.S.A.).

### Bacterial Strains, Media, and Growth Conditions

MRSA strain XN108 was isolated from Southwest Hospital (Chongqing, China) (Zhang et al., 2013), and *Pseudomonas aeruginosa* strain PA1 (Lu et al., 2015; Li et al., 2016a; Li et al., 2016b) was isolated from Xinqiao Hospital (Chongqing, China), and they were both kept in our laboratory. Methicillin-sensitive *S. aureus* (MSSA) strain ATCC 25923, *P. aeruginosa* strain PAO1, and *Acinetobacter baumannii* strain AB1 were all purchased from China Center for Type Culture Collection. All strains were grown in brain heart infusion (BHI) broth medium with constant shaking overnight at 37°C. Phage P108, which encodes the LysP108 endolysin, was isolated from hospital sewage using the MRSA strain XN108 as host bacterium. For transformation, *E. coli* strains DH5α and BL21 (DE3) were prepared for cloning and recombinant protein expression, respectively, and they were cultured in Luria-Bertani (LB) broth at 37°C.

### Bioinformatics Analysis of LysP108

The phage P108 genome was sequenced and submitted to the GenBank database (accession number: NC025426). The amino acid sequence of LysP108 was analyzed using the Basic Local Alignment Search Tool (BLAST, http://www.ncbi.nlm.nih.gov/BLAST/) for comparison. Subsequently, based on the protein sequence, the 3D structure of LysP108 was predicted using the Swiss-Model tool (http://swissmodel.expasy.org). The amino acid sequences of LysGH15 (protein ID: ADG26756.1) (Zhang et al., 2018), LysK (protein ID: AAO47477.2) (Fujita et al., 2017), HydH5 (protein ID: ACJ64586.1) (Rodriguez et al., 2011), PlyGRCS (protein ID: AHJ10590.1) (Linden et al., 2015), CF-301 (protein ID: ZP_03625529.1) (Schuch et al., 2014), endolysin of *Listeria* phage vB_LmoS_293 (protein ID: AJE28090.1) (Pennone et al., 2019), PlyAB1 (protein ID: YP_008058242.1) (Huang et al., 2014), lysostaphin of *Nocardia seriolae* (protein ID: APB01676.1) (Jayakumar et al., 2020) were downloaded from the National Center for Biotechnology Information (NCBI) Protein Database (https://www.ncbi.nlm.nih.gov/protein). Sequence homology analysis of LysP108, LysGH15, LysK, HydH5, PlyGRCS, CF-301, endolysin of *Listeria* phage vB_LmoS_293, PlyAB1, and lysostaphin of *Nocardia seriolae* was performed using the software DNAMAN (https://www.lynnon.com/dnaman.html).

### Cloning, Overexpression, and Purification of LysP108

Recombinant LysP108 was produced through the *E. coli* expression system. The gene fragment encoding LysP108 was amplified by PCR with a set of specific primers (forward: TAAGAAGGAGATATACATATGAAAAAAAAAGATAAACGTGGTAAGAAACC and reverse: TGGTGGTGCTCGAGTGCGGCCGCTTTGAATACTCCCCAAGCAA, with the NdeI/NotI restriction endonuclease sites underlined). The PCR amplification condition consisted of initial denaturation step at 95°C for 2 min, followed by 35 cycles of denaturation step at 95°C for 20 s, annealing at 52°C for 20 s, and elongation at 72°C for 30 s, and final extension step at 72°C for 5 min. After double enzyme digestion, the PCR product and pET21a vector were linked together by using Gibson Assembly at 50°C for 30 min to construct the recombinant plasmid pET21a-LysP108. DNA sequences were verified for the constructed plasmid. Next, C-terminal six-His-tagged LysP108 plasmid was transformed into *E. coli* BL21 (DE3) to screen the positive clones.

A single positive colony was cultured in LB broth added with 100 μg mL^-1^ ampicillin at 37°C under constant shaking at 180 rpm. When the OD_600_ value of the bacterial fluid reached approximately 0.6, isopropyl β-D-1-thiogalactopyranoside (IPTG) was added to reach the working concentration of 0.1 mM. After incubation for 10 h at 23°C, the bacterial cells were collected by centrifugation and disrupted by ultrasonication, followed by centrifugation at 10,000 g for 30 min to remove the bacterial fragments. Then, the supernatant containing soluble protein was filtrated through a 0.4 μm filter membrane. The protein was purified with nickel affinity chromatography. The purity and size of the obtained protein were confirmed with sodium dodecyl sulfate-polyacryl amide gel electrophoresis (SDS-PAGE), in which the gel was stained by Coomassie brilliant blue. Then the protein concentration was detected by a bicinchoninic acid assay. The LysP108 solution was dialyzed against 10 mM PBS containing 20% glycerol and stored at -20°C.

### Determination of LysP108 Antibacterial Activity

The antibacterial activity of LysP108 was determined by colony forming unit (CFU) reduction assay as previously described. (Dong et al., 2015) All bacteria strains were cultured to early log phase, harvested, and resuspended in the same volume of 20 mM Tris-HCl buffer (pH 7.5). For Gram-negative bacteria, early log phase cells were pelleted and resuspended in 20 mM Tris-HCl buffer supplemented with 0.1 M EDTA for 5 min at room temperature (RT). EDTA at high concentrations is toxic to the cells. Thus, the outer membrane was treated only for 5 min. Then, cells were pelleted and washed with 20 mM Tris-HCl buffer thrice to remove the remaining EDTA. Next, 20 µL of LysP108 (1 mg mL^-1^) was added to 80 µL of resuspended bacteria and incubated for 30 min at 37°C. Afterward, the mixture was serially diluted by 10-fold and plated on BHI agar plates. The CFU was calculated after 24 h of incubation at 37°C to determine the number of viable cells. The above tests were repeated for three times, and the mean values were calculated. The final CFU was obtained by multiplying the average number of colonies on the plate by the dilution factor.

Factors affecting LysP108 lytic activity were analyzed using early log phase bacteria under different reaction conditions, as follows: LysP108 concentration of 0–1000 μg mL^-1^; pH of 4.0–12.0; and temperature of 30°C–90°C. To assess the effect of LysP108 concentration on lytic activity, various concentrations (0–1000 μg mL^-1^) of LysP108 and the MRSA strain XN108 cell suspensions were mixed thoroughly and incubated for 30 min at 37°C. To test the pH stability, 250 μg mL^-1^ of LysP108 was added to the bacterial cell suspension in the following buffers: 20 mM sodium acetate for pH 4.0–5.0; 20 mM Tris-HCl for pH 6.0–8.0; 20 mM glycine for pH 9.0–10.0; and 20 mM sodium carbonate for pH 11.0–12.0. Briefly, 80 μL bacterial resuspended solution at different pH was mixed with 20 μL LysP108 and incubated for 30 min at 37°C, while the control group was treated with bacterial resuspended solution at different pH and 20 μL PBS. To test the thermal stability, 250 μg mL^-1^ of LysP108 was incubated at different temperatures (30°C–90°C) for 10 min. After cooling to room temperature, 80 μL bacterial solution was mixed with 20 μL LysP108 treated at different temperatures and incubated at 37°C for 30 min, while the control group was treated with bacterial solution and 20 μL PBS without temperature treatment. All experiments were repeated thrice. The values were the means and standard deviations from triplicate assays.

### Assessment of LysP108 Lytic Activity

The interactions between LysP108 and MRSA (XN108 and SCC*mec* V) strains were assessed *in vitro*. LysP108 and bacteria cells were suspended in 10 mM PBS (pH 7.4). Then, 200 μL of LysP108 (250 μg mL^-1^) and 800 μL of the test bacteria (10^8^ CFU mL^-1^) were mixed together and incubated at 37°C for 5, 30, and 60 min, respectively. For the complete removal of free LysP108, the mixtures were then centrifuged at 5000 g for 5 min and washed thrice by PBS (10 mM; pH 7.4). Subsequently, the sediments comprising bacteria with LysP108 were suspended in PBS (10 mM; pH 7.4). The morphology of aggregated pellets were detected under a scanning electron microscope (SEM, Inspect F, Philips, The Netherlands).

### *In Vitro* Live/dead Bacteria Staining Assay

Live/dead staining assay was performed by using a live/dead bacteria staining kit. The kit contained two fluorescent dyes, SYTO 9 and PI. The green SYTO 9 dye entered both intact bacteria and bacteria with damaged cell structure. However, the red PI dye only entered bacteria with the damaged cell membrane or wall. Briefly, 2 mL of MRSA strain XN108 was cultured to post log phase, harvested, and resuspended in 1 mL of 0.85% NaCl. Then, 200 µL of LysP108 (250 μg mL^-1^) was added into 800 µL of resuspended bacteria and incubated for 1 h at 37°C with 100 rpm. The above mixture was centrifuged at 5000 g for 5 min and suspended in 0.85% NaCl. Next, the suspensions were stained with live/dead dye solution (1.5 µL of SYTO 9 and 1.5 µL of PI) simultaneously for 15 min in the dark. Then, the stained bacteria were washed with 0.85% NaCl twice and observed under a fluorescent microscope.

### Crystal Violet Staining Assay

Crystal violet staining assay was performed as previously described with some modifications. (Wu et al., 2003) The MRSA strain XN108 was incubated overnight in BHI culture medium, after which it was prepared and sub-cultured in a 96-well polystyrene microplate with the same culture medium. After incubating the microplate for 24 h at 37°C, all wells were washed with 10 mM PBS (pH 7.4). Once the biofilm was formed, the experimental group wells were filled with 100 µL of LysP108 (250 µg mL^-1^). PBS at 10 mM was used as the negative control. After incubation for 5 h at 37°C with 100 rpm, each well was washed once with 10 mM PBS and stained with 0.1% crystal violet for 10 min at RT. Washing with 10 mM PBS was repeated thrice, and solubilization with 33% acetic acid was performed. The absorbance of the obtained solution was detected with a plate reader and measured at 570 nm (OD_570_).

### Standard Checkerboard Broth Micro-Dilution Assay

A standard checkerboard broth micro-dilution assay was used to test whether there is a synergistic interaction between LysP108 and vancomycin. Briefly, different dilutions of vancomycin and LysP108 were incubated vertically and horizontally with a bacterial inoculum of 5 × 10^5^ CFU per well in a final volume of 50 µL, respectively. MRSA strain XN108 was used to test the interaction between endolysin and vancomycin. The plates were incubated at 37°C with gentle shaking and the bacterial growth rate was determined by reading OD_600_ for 20 h. The fractional inhibitory concentration of antibiotic and LysP108 was plotted as an isobologram.

### *In Vivo* Animal Experiments

To study the antibacterial effect of LysP108 *in vivo*, a subcutaneous abscess model on male BALB/c mice (6–8 weeks old; 20–25 g in weight) was established. Mice were anesthetized with an intraperitoneal injection of 1% pentobarbital at a dose of 50 mg kg^-1^, and the dorsal surface of mice was shaved and cleaned with 75% alcohol. Subsequently, 100 μL of MRSA suspension (1 × 10^8^ CFU mL^-1^) was injected subcutaneously into the right and left sides of the shaved back of each test mouse. After 24 h, a focal MRSA infection formed as a subcutaneous abscess.

For the endolysin group, the infected mice were injected subcutaneously with 100 μL of 250 μg ml^-1^ of LysP108, and for the vancomycin group, vancomycin solution was injected intravenously at a dose of 20 mg kg^-1^. In addition, for the co-administration group, 100 μL of 250 μg ml^-1^ LysP108 was injected subcutaneously, and at the same time, the vancomycin solution was injected intravenously (20 mg kg^-1^). PBS at 100 μL was injected subcutaneously as the control treatment. Except for vancomycin that was administered once every 12 h, LysP108 and PBS were administered once every 24 h for 10 days.

Using standard circular paper (blue) with a diameter of 6 mm as a reference, the mice were photographed, and observations were recorded daily. Meanwhile, the abscess area was calculated and analyzed through Image J (NIH) software to draw the abscess area curve of the skin infection on the back of the mice. After being treated with paraffin embedding, serial section, and hematoxylin and eosin (H&E) staining, the samples were histologically examined under an optical microscope.

### Statistical Analysis

Data in line with normal distribution were presented as mean ± standard deviation (SD) of at least 3 or more independent measurements. Statistical analysis was performed by two-tailed Student’s *t* test or one-way ANOVA using SPSS 18.0 (IBM, USA). Bonferroni *post hoc* test was used for multiple *post hoc* comparisons to determine statistical significance after one-way ANOVA. *P* < 0.05 was considered significant. Graph analysis was performed using GraphPad Prism 8.0 (GraphPad Software, USA).

## Results

### LysP108 Sequence Analysis and Protein Purification

**Figure 1:**
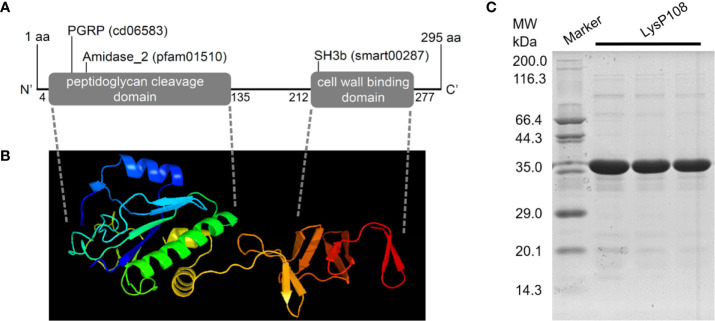
Characterization and purification of endolysin LysP108. **(A)** Schematic illustration of structure of phage endolysin LysP108. **(B)** 3D structure of endolysin protein LysP108. **(C)** SDS-PAGE photograph of purified protein LysP108.

Genomic annotation revealed that the phage P108 encode a putative endolysin, which was named LysP108 (GenBank protein ID: YP_009099525.1). In silico analysis of LysP108 predicted a 295-amino acid protein (~34 kDa) with two independent domains: the peptidoglycan cleavage domain (PCD) and cell wall binding domain (CBD) (**Figure 1A**). In general, PCD is responsible for cleaving specific peptidoglycan covalent bond structures, whereas CBD is responsible for adhering to the bacterial cell wall. The predicted sites of PCD ranged from 4 to 135 amino acids, and those of CBD ranged from 212 to 277 amino acids. To study the tertiary structure of endolysin LysP108, Swiss-Model homology modeling was used to construct a 3D structure. PCD and CBD were predicted to be folded independently, whereas amidase-2 was folded together with PCD, thereby enhancing the lytic activity of PCD (**Figure 1B**). Sequence homology analysis revealed that although LysP108 is homologous with several endolysins identified previously, it also has differences in the amino acid sequence and domain composition (**Figure S1**).

Sequences encoding LysP108 with His-tag at the C-terminus were successfully constructed into IPTG-inducible expression plasmid pET21a. Thus, the recombinant plasmid was named as pET21a-LysP108 (**Figure S2**). LysP108 was then expressed in *E. coli* cells bearing pET21a-LysP108 as a soluble form after it was induced by IPTG at 37°C (**Figure S3**). The purified protein showed the right mass and near homogeneity (~34 kDa), as revealed by SDS-PAGE (**Figure 1C**).

### LysP108 Showed Lytic Activity to MRSA Strain XN108

**Figure 2:**
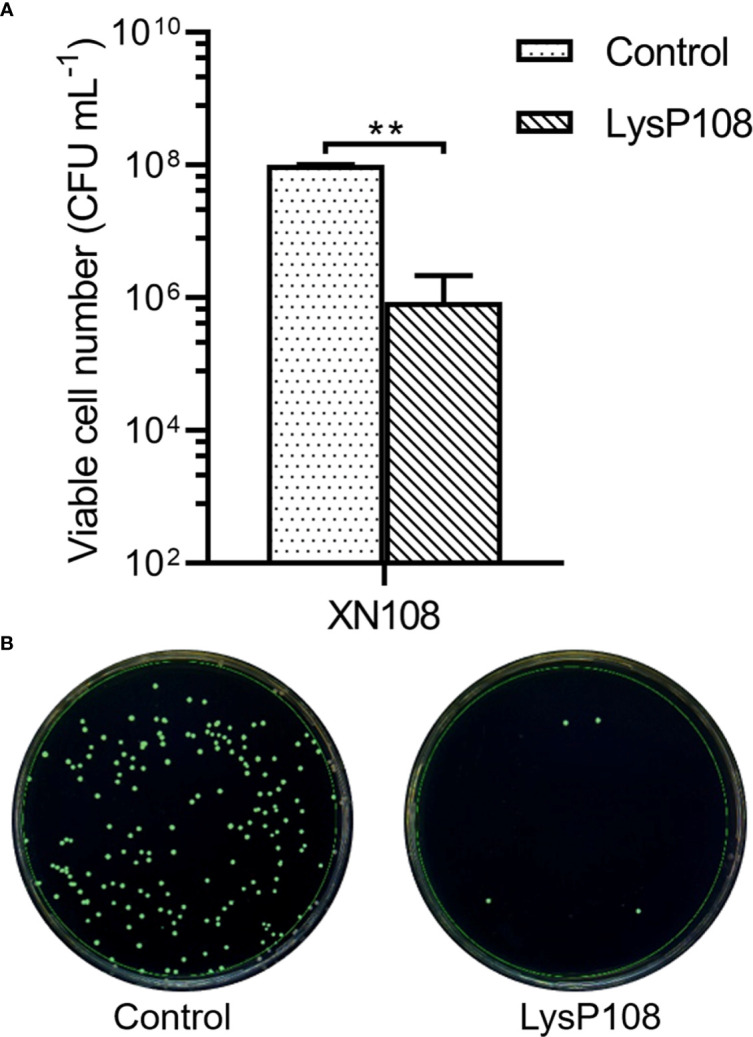
Lytic activity of LysP108. **(A)** Identification of the lytic activity of endolysin LysP108 on XN108 by CFU reduction assay. **(B)** Scanning diagram of plate colony. (n=3). **P < 0.01.

The lytic activity of LysP108 was determined through a CFU reduction assay with MRSA strain XN108 as the target. LysP108 induced XN108 lysis, and the viable cell significantly decreased by approximately 2 log units after 30 min of treatment (**Figure 2A**). Meanwhile, through the automatic colony counter, the scanning diagram of plate colony directly reflected the significant decrease in the number of viable bacteria (**Figure 2B**). The above results demonstrated that endolysin LysP108 can directly lyse its host bacteria XN108 *in vitro*.

### Influence of Concentration, pH, and Temperature on LysP108 Antibacterial Activity

**Figure 3:**
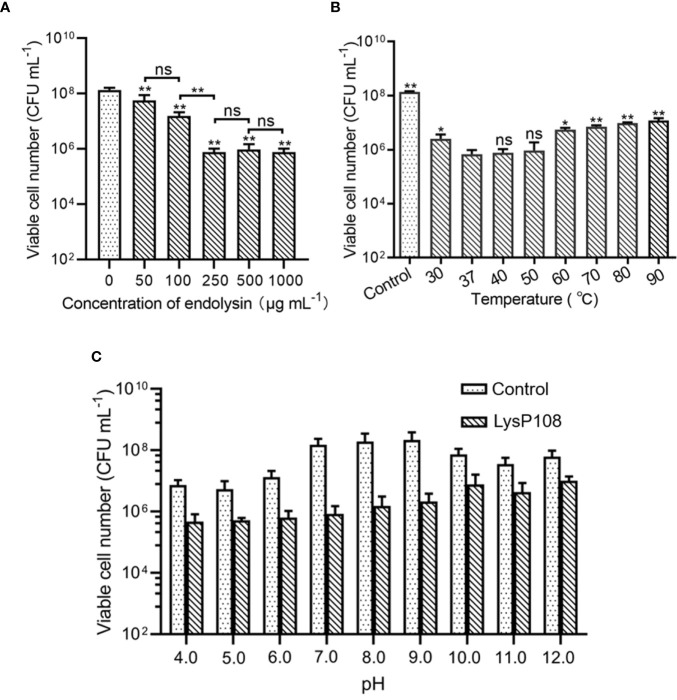
Determination of the optimum conditions for the lytic activity of endolysin LysP108: **(A)** concentration (n=3), **(B)** temperature (n=3), and **(C)** pH (n=3). *P < 0.05, **P < 0.01, and ns, not significant.

To assess the effect of concentration on the lytic activity of LysP108, different concentrations (0–1000 μg mL^-1^) of LysP108 were tested. The addition of 250 µg mL^-1^ LysP108 reduced the viable numbers of XN108 by 2 log units, which was the most significant reduction. At a concentration greater than 250 µg mL^-1^, the effect of LysP108 reached saturation, indicating that the optimal working concentration was 250 µg mL^-1^ (**Figure 3A**).

The result showed that LysP108 was highly active at the temperature range of 37°C–50°C, but its activity significantly decreased after heat treatment at 60°C (**Figure 3B**). Considering that the temperature of the human body is approximately 37 °C in practical applications, the optimum temperature of LysP108 is determined to be 37 °C.

Lytic activity analysis at different pH values demonstrated that the low pH of the reaction buffer significantly affected cell viability, whereas relatively high lytic activity was observed at pH 7.0, with a reduction of 2 log units of viable cells compared with the control (no LysP108 addition), as shown in **Figure 3C**. Thus, the optimum pH value of LysP108 was set at pH 7.0. All data further confirmed the antibacterial activity of LysP108.

### The Antibacterial Spectrum of LysP108

**Figure 4:**
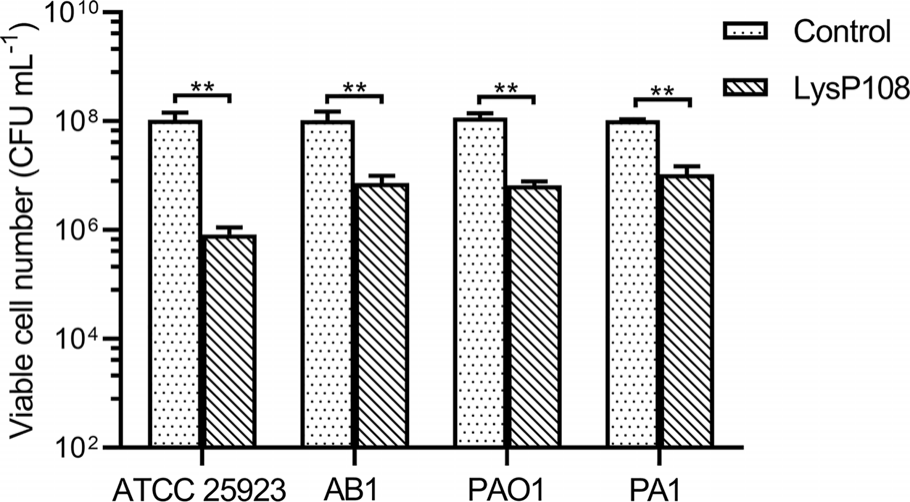
The antibacterial activity of endolysin LysP108 toward *S. aureus*, *P. aeruginosa*, and *A. baumannii*. (n=3). **P < 0.01.

Just like most of the reported phage derived endolysins, LysP108 could not lyse Gram-negative bacteria directly (data not shown), which was due to the protection of the outer membrane. EDTA was used to treat *P. aeruginosa* strains PAO1 and PA1 and *A. baumannii* strain AB1 to remove the outer membrane before the addition of LysP108. As shown in **Figure 4**, LysP108 was able to lyse all three Gram-negative strains and reduced the viable cells by 1 log unit. Therefore, LysP108 was active against Gram-negative bacteria without outer membrane. Besides, the viability of *S. aureus* strain (ATCC 25923) was reduced by 2 log units after incubation with LysP108 for 30 min (**Figure 4**).

### *In Vitro* Bactericidal Effect of LysP108

**Figure 5:**
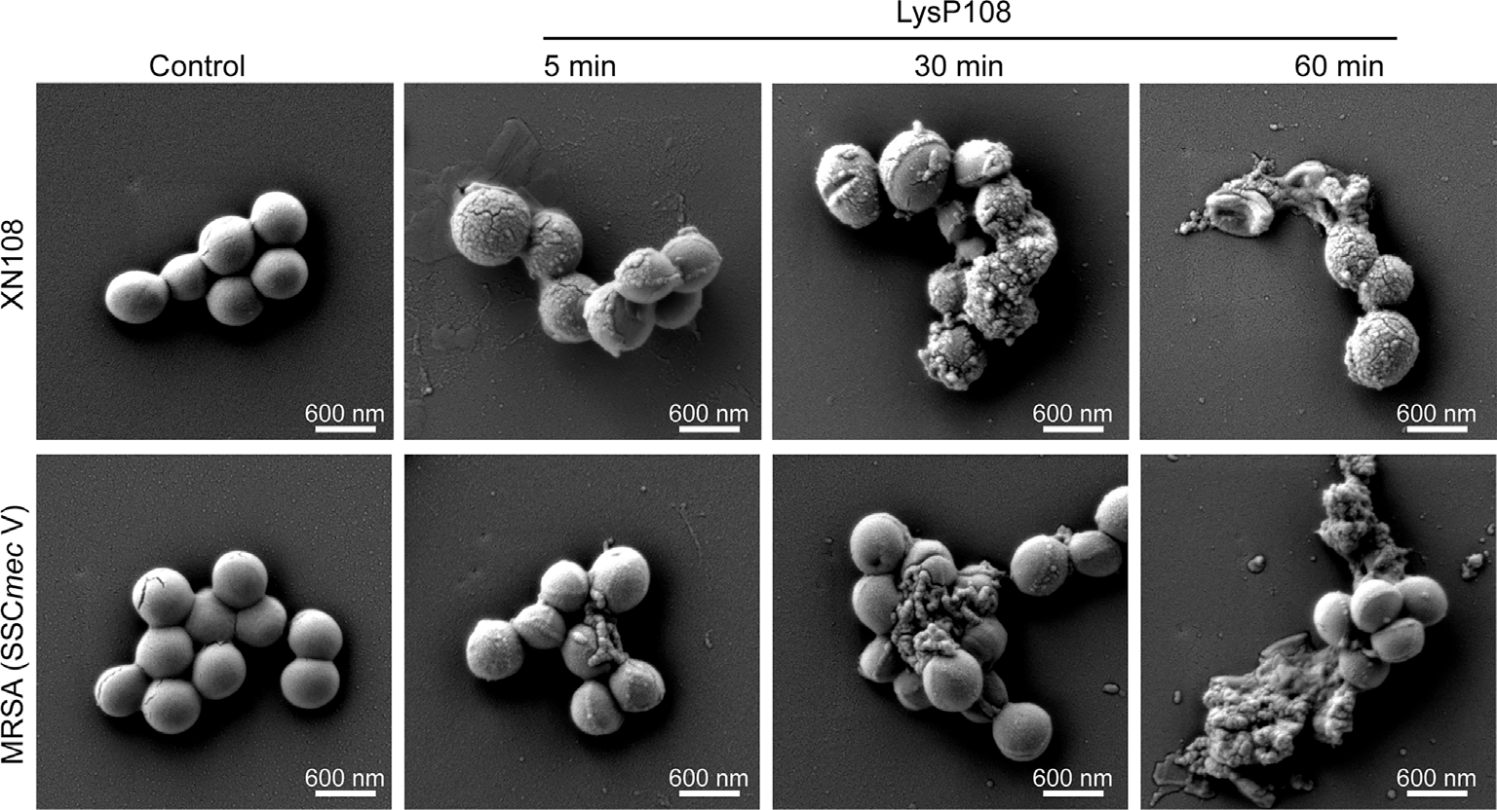
SEM micrographs of MRSA strains (XN108 and SCC*mec* V) after incubation with LysP108 and PBS for different durations.

**Figure 6:**
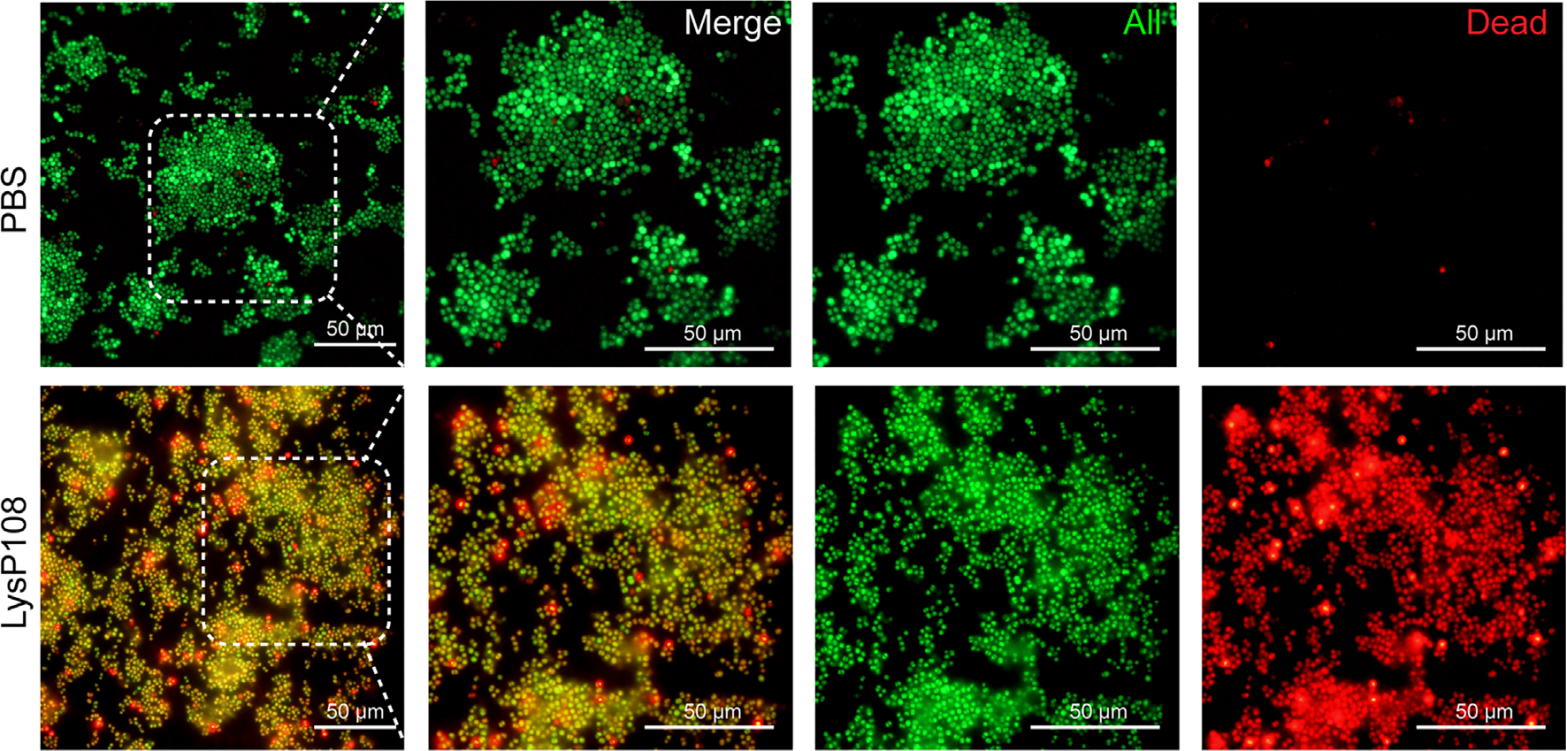
Fluorescent micrographs of live/dead bacteria staining. Green, all bacteria; red, dead bacteria.

Supporting evidence for the lytic property of LysP108 was provided in the SEM analysis. As shown in **Figure 5**, after incubation with LysP108 for different durations, MRSA (XN108 and SCC*mec* V) cells were gradually lysed *in vitro*. The bactericidal effect of endolysin was further verified using a live/dead bacteria staining kit, and the results were observed using a fluorescent microscope. As shown in **Figure 6**, the green fluorescent channel showed all bacteria, whereas the red fluorescent channel only showed the dead bacteria. The two fluorescent signals were coincident, indicating that most bacteria were killed under the effect of LysP108. The bactericidal rate was calculated as approximately 90%.

### Biofilm Reduction Activity of LysP108

**Figure 7:**
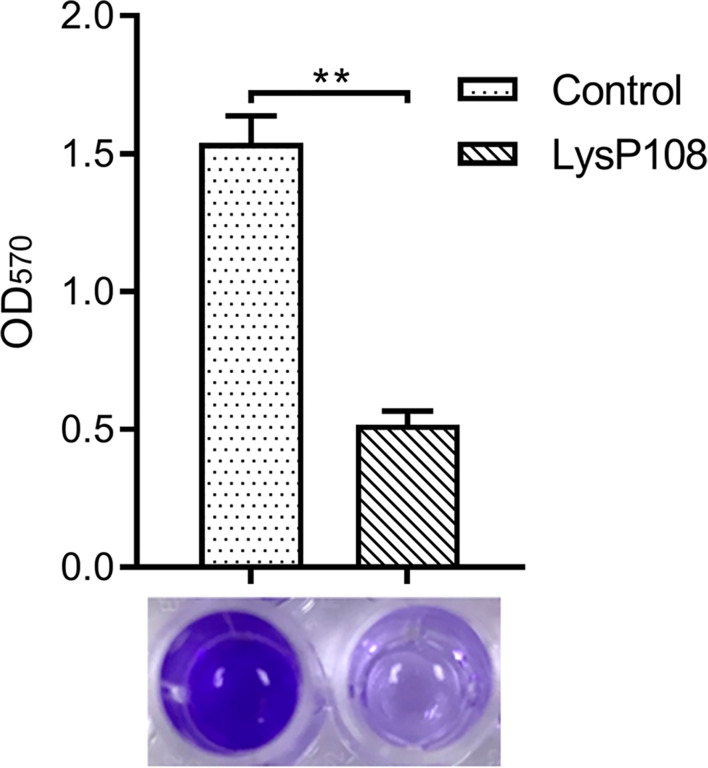
Identification of the disruption ability of endolysin LysP108 toward bacterial biofilm by crystal violet staining. (n=3). **P < 0.01.

The biofilm matrix disruption by LysP108 was measured by plate reader at OD_570_ value and further verified by visual comparison in crystal violet-staining. As shown in **Figure 7**, the crystal violet-stained color was significantly reduced in the LysP108-treated group compared with the PBS-treated control group. Approximately 66% of the biofilm was successfully removed in the LysP108-treated group compared with the control, indicating that LysP108 had strong anti-biofilm activity.

### Synergistic Antibacterial Activity of LysP108 and Vancomycin *In Vivo*

**Figure 8:**
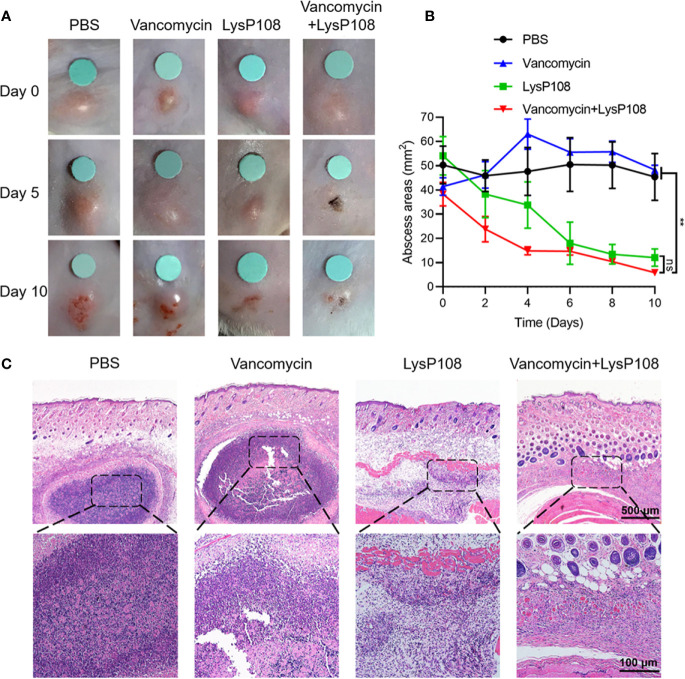
Synergistic antibacterial effect of LysP108 and vancomycin *in vivo*. **(A)** Photographs of endolysin combined with vancomycin for the treatment of mice subcutaneous abscess. **(B)** Subcutaneous abscess area curve of MRSA infected on the back of mice. (n=6). **(C)** Representative H&E staining images of infected skin subjected to various treatments. The violet circles indicate the subcutaneous abscess. Scale bar: 100 μm. **P < 0.01; ns, not significant.

After abscesses formed on the subcutaneous tissues of mice, the subcutaneously infected abscesses were treated with vancomycin, LysP108, vancomycin combined with LysP108, and PBS. The mice were photographed, and observations were recorded daily. After 10 days of treatment, no obvious abscess was found in mice in the endolysin group and the co-administration group, whereas skin abscess still obviously existed and was accompanied by severe tissue ulceration in the vancomycin and control groups (**Figure 8A**). The poor therapeutic effect of vancomycin alone was due to the intermediate resistance of MRSA strain XN108 to vancomycin. A synergistic antibacterial effect existed between LysP108 and vancomycin, which indicated that the presence of endolysin may enhance the sensitivity of XN108 to vancomycin (**Figures 8B** and **S4**). Furthermore, H&E staining results suggested that obvious abscess and inflammatory reaction occurred in the vancomycin and control groups, but not in the combined therapy group (**Figure 8C**).

## Discussion

Over the recent years, resistance to antimicrobial drugs has become a growing global concern (Tornimbene et al., 2018). One of the most alarming antibiotic-resistant bacterial species is *S. aureus*. Specifically, MRSA are the group of *S. aureus* strains resistant to virtually all classes of antibiotics and lead to severe and life-threatening infections (Brown et al., 2021). Therefore, there is an urgent need to develop effective therapeutic agents against MRSA (Scallan et al., 2011). Among many antimicrobial agents against *S. aureus*, bacteriophage endolysins have been found promising because of their broad activity spectrum, rapid antibacterial activity, and low probability for developing resistance (Toyofuku et al., 2019). Several endolysins identified from genomes of bacteriophages have been applied exogenously in the form of purified recombinant proteins, which can induce lysis and death of Gram-positive bacterial cells (Rodriguez et al., 2011; Schuch et al., 2014; Linden et al., 2015; Fujita et al., 2017; Zhang et al., 2018). However, these endolysins are still far from clinical applications, so more endolysins should be explored. In this study, both *in vitro* and *in vivo* assays revealed that LysP108 had potential application value to combat antibiotic-resistant bacteria, which lay a good foundation for further study to improve LysP108.

Generally, the structure of endolysins that act on Gram-positive cell walls consist of one or more PCDs with catalytic activity linked to CBD with binding activity. LysP108 employs a typical two-domain modular architecture consisting of an N-terminal PCD domain and a C-terminal CBD domain, encoding a 295-amino acid protein with a deduced molecular mass of 34 kDa (**Figure 1**). Phage derived endolysins can be classified in three groups based on their cleavage specificity: (1) endopeptidases – targeting peptide bonds, including L-alanoyl-D-glutamate endopeptidase and interpeptide bridge endopeptidase; (2) amidases – targeting amide bonds, including the N-acetylmuramoyl-L-alanine amidases; (3) glycosidases – targeting glyosidic bonds, including transglycosylases, N-acetyl-β-D-glucosaminidases, and N-acetyl-β-D-muramidases (Kashani et al., 2018). For LysP108, it contains an amidase domain at the N-terminal, which indicates that it belongs to the second group of N-acetylmuramoyl-L-alanine amidases. A number of studies have demonstrated the increased lytic activity of several enzymes upon deletion of their binding domains (Dunne et al., 2014; Singh et al., 2014). Hence, the results of this study can be served as the basis for the modification of LysP108, such as using only the catalytic domain instead of the full-length endolysin to enhance its activity.

Previous studies have shown that the stability of endolysin is critical to its application value (Kashani et al., 2018). LysP108 was highly active under a diverse range of pH and was tolerated to different temperatures. However, its optimal working concentration was 250 µg mL^-1^, which was a disadvantage relative to the small dosage of most antibiotics (Wilm et al., 2021). The antibacterial activity of LysP108 was confirmed by its ability to reduce the viability of MRSA strain XN108 (**Figure 2**). As shown in **Figure 5**, LysP108 disintegrated the cell wall of MRSA externally in a time-dependent manner, which eliminated the antibacterial activity of other pathways. This observation was quite similar with the typical phenomenon of osmotic-mediated cell lysis following the actions of phage lysins against Gram-positive bacteria reported elsewhere (Linden et al., 2015; Kashani et al., 2018).

Although LysP108 showed good antibacterial activity against other tested Gram-positive bacterial cells, including MSSA strain (ATCC 25923), it cannot directly destroy the cell wall of Gram-negative bacteria. For Gram-negative bacteria with outer membrane that prevents the entry of endolysin into the cell wall (Nikaido, 2003), EDTA was used as a pre-treatment to disrupt the outer membrane (Dong et al., 2015). Subsequently, LysP108 had antibacterial activity against the tested Gram-negative bacterial cells that have been pre-treated with EDTA, including *P. aeruginosa* strains PAO1 and PA1 and *A. baumannii* strain AB1 (**Figure 4**). However, the reagents used to treat the outer membrane are toxic and cannot be used in clinical treatment. Several reports have shown the feasibility of trans-membrane modification (Briers et al., 2014), so it is expected that LysP108 could be modified in future study to act directly on Gram-negative bacteria.

Furthermore, LysP108 showed a 66% reduction in OD_570_ after crystal violet staining compared with the untreated control (**Figure 7**), thereby presenting a notable disrupting activity against biofilms formed by MRSA. LysP108 possibly lyses individual staphylococcal cells embedded in the extracellular matrix of the biofilm, resulting in destabilization of the biofilm and its detachment from the surface, as suggested by previous studies (Shen et al., 2013). Therefore, LysP108 could be used to treat infections, such as osteomyelitis, periodontitis, and chronic rhinosinusitis (Archer et al., 2011), which are caused by biofilm-forming *S. aureus* cells.

A considerable body of evidence has revealed that MRSA infection remains one of the main causes of hospital infections, leading to increasing rates of morbidity and mortality (Borg et al., 2021). Besides, community-acquired MRSA typically leads to superficial skin infections that can ultimately progress to induce severe invasive complications, such as necrotizing fasciitis (Takadama et al., 2020). Therefore, the selection of the skin abscess model of MRSA infection was representative. *In vivo* animal experiments revealed that the area of subcutaneous abscess of mice infected with MRSA was significantly reduced after the combined injection of LysP108 and vancomycin in comparison with monotherapy (**Figure 8**). Moreover, the standard checkerboard broth micro-dilution assay indicated that there is a synergistic interaction between LysP108 and vancomycin (**Figure S4**). When the concentration of vancomycin was 8 µg mL^-1^ and the concentration of LysP108 was greater than 100 µg mL^-1^, the growth of bacteria could be inhibited (**Figure S4**). The standard broth microdilution method had been employed to measure susceptibility of MRSA strain XN108 toward vancomycin and the MIC of vancomycin was 16 μg mL^-1^ (Zhang et al., 2013; Wang et al., 2020). Likewise, the combination of endolysin CF-301 and daptomycin for the treatment of bacteremia caused by MRSA infection significantly improved the survival rate of mice compared with the individual application of these two antibacterial substances (Schuch et al., 2014). Thus, endolysin LysP108 is expected to be used in combined therapy, although more antibiotics should be tried in the future.

In conclusion, our present study demonstrated the antibacterial effect of endolysin LysP108 *in vitro* and *in vivo*, and showed the potential of LysP108 in combination with antibiotics, suggesting a new way for solving the increasingly serious problem of drug resistance in Gram-positive bacteria.

## Data Availability Statement

The datasets presented in this study can be found in online repositories. The names of the repository/repositories and accession number(s) can be found in the article/**Supplementary Material**.

## Ethics Statement

The animal study was reviewed and approved by Laboratory Animal Welfare and Ethics Committee of Third Military Medical University (Army Medical University), 30# Gaotanyan St., Shapingba District, Chongqing 400038, China.

## Author Contributions

ZF and SL conceived the study. YL and YW performed the experiments. JW, YZ, and QZ analyzed the data. YL, YW, and GL wrote the paper. All authors contributed to the article and approved the submitted version.

## Funding

This work was supported by the National Natural Science Foundation of China (grant 31871251 to SL; grant 21775125 to ZF) and the Natural Science Foundation of Chongqing (grant cstc2018jcyjAX0175 to ZF). The funders had no role in study design, data collection and interpretation, or the decision to submit the work for publication.

## Conflict of Interest

The authors declare that the research was conducted in the absence of any commercial or financial relationships that could be construed as a potential conflict of interest.

The reviewer JX declared a shared affiliation, with no collaboration, with one of the authors, ZF, to the handling editor at the time of review.
