# Supplementary material for: Phage Endolysin LysP108 Showed Promising Antibacterial Potential Against Methicillin-resistant Staphylococcus aureus
Source: Front Cell Infect Microbiol. 2021 Apr 15;11:668430. doi: 10.3389/fcimb.2021.668430 (PMC8082462; doi:10.3389/fcimb.2021.668430)
Supplement: Supplementary file 1 [file DataSheet_1.pdf]

Supplementary Material

Phage Endolysin LysP108 Showed Promising Antibacterial Potential Against Methicillin-resistant *Staphylococcus aureus*

Yifei Lu[1,†], Yingran Wang[1,#], Jing Wang[‡], Yan Zhao[‡], Qiu Zhong[$], Gang Li[‡], Zhifeng Fu[,‖] and Shuguang Lu[*,‡]*

[†]Institute of Burn Research, Southwest Hospital, State Key Lab of Trauma, Burn and Combined Injury, Army Medical University, Chongqing 400038, China.

[‡]Department of Microbiology, College of Basic Medical Science, Army Medical University, Chongqing 400038, China.

[‖]College of Pharmaceutical Sciences, Southwest University, Chongqing 400716, China.

[#]Department of clinical laboratory medicine, Southwest Hospital, Army Medical University, Chongqing 400038, China.

[$]Department of clinical laboratory medicine, Daping hospital, Army medical university.

**Corresponding author. Tel.: +86-23-6825-0184; Fax: +86-23-6825-1048.*

**Corresponding author. Tel.: +86-23-6875-2243; Fax: +86-23-6877-1353.*

E-mail address: fuzf@swu.edu.cn (Z.F. Fu); shulang88@126.com (S.G. Lu).

[1]The authors contributed equally to this work.

Table of Contents

Figure S1 ·········· S3, S4

Figure S2 ·········· S5

Figure S3 ·········· S6

Figure S4 ·········· S7

```
LysP108_[Staphylococcus_phage_P108]                ........................................    0
LysGH15_[Staphylococcus_phage_GH15]                MAKTQAEINKRLDAYAKGTVDSPYRIKKATSYDPSFGVME   40
LysK_[Staphylococcus_phage_K]                      MAKTQAEINKRLDAYAKGTVDSPYRVKKATSYDPSFGVME   40
HydH5_[Staphylococcus_phage_vB_SauS-phiIPLA88]     ..............MGLPNPKDRKPTASEVVEWALYMAKN   26
PlyGRCS_[Staphylococcus_phage_GRCS]                ........................................    0
CF-301_[prophage_of_Streptococcus_suis_89/1591]    ........................................    0
endolysin_[Listeria_phage_vB_LmoS_293]             ........................................    0
PlyAB1_[Acinetobacter_phage_Abp1]                  ........................................    0
Lysostaphin_[Nocardia_seriolae]                    ........................................    0
Consensus

LysP108_[Staphylococcus_phage_P108]                ........................................    0
LysGH15_[Staphylococcus_phage_GH15]                AGAIDADGYYHAQCQDLITDYVLWLTDNKVRTWGNAKDQI   80
LysK_[Staphylococcus_phage_K]                      AGAIDADGYYHAQCQDLITDYVLWLTDNKVRTWGNAKDQI   80
HydH5_[Staphylococcus_phage_vB_SauS-phiIPLA88]     RRVIDVDRSYGGQCWDVPNYILERYWGFRTWGNANAMAQK   66
PlyGRCS_[Staphylococcus_phage_GRCS]                ........................................    0
CF-301_[prophage_of_Streptococcus_suis_89/1591]    ........................................    0
endolysin_[Listeria_phage_vB_LmoS_293]             ........................................    0
PlyAB1_[Acinetobacter_phage_Abp1]                  ........................................    0
Lysostaphin_[Nocardia_seriolae]                    ........................................    0
Consensus

LysP108_[Staphylococcus_phage_P108]                ........................................    0
LysGH15_[Staphylococcus_phage_GH15]                KQSYGTGFKIHENKPSTVPKKGWIAVFTSGSYQQWGHIGI  120
LysK_[Staphylococcus_phage_K]                      KQSYGTGFKIHENKPSTVPKKGWIAVFTSGSYEQWGHIGI  120
HydH5_[Staphylococcus_phage_vB_SauS-phiIPLA88]     SNYRGRDFKIYRNTASFTPKPGDWAVWAN...RNPGHVAI  103
PlyGRCS_[Staphylococcus_phage_GRCS]                ........................................    0
CF-301_[prophage_of_Streptococcus_suis_89/1591]    ........................................    0
endolysin_[Listeria_phage_vB_LmoS_293]             ........................................    0
PlyAB1_[Acinetobacter_phage_Abp1]                  ........................................    0
Lysostaphin_[Nocardia_seriolae]                    ........................................    0
Consensus

LysP108_[Staphylococcus_phage_P108]                ........................................    0
LysGH15_[Staphylococcus_phage_GH15]                VYDGGNTSTFTILEQNWN..GYANKKPTKRVDNYY....G  154
LysK_[Staphylococcus_phage_K]                      VYDGGNTSTFTILEQNWN..GYANKKPTKRVDNYY....G  154
HydH5_[Staphylococcus_phage_vB_SauS-phiIPLA88]     VVGPADKNAFVSVDQNWYTANWSGSPPYKIKHTYHDGPGG  143
PlyGRCS_[Staphylococcus_phage_GRCS]                ........................................    0
CF-301_[prophage_of_Streptococcus_suis_89/1591]    ........................................    0
endolysin_[Listeria_phage_vB_LmoS_293]             ........................................    0
PlyAB1_[Acinetobacter_phage_Abp1]                  ........................................    0
Lysostaphin_[Nocardia_seriolae]                    ........................................    0
Consensus

LysP108_[Staphylococcus_phage_P108]                ........................................    0
LysGH15_[Staphylococcus_phage_GH15]                LTHFIEIPVKAGTTVKKET....AKKSASKTPAPKKKATL  190
LysK_[Staphylococcus_phage_K]                      LTHFIEIPVKAGTTVKKET....AKKSASKTPAPKKKATL  190
HydH5_[Staphylococcus_phage_vB_SauS-phiIPLA88]     VTHFVRPPYHPDKTTPAPQPVPKPKDDSDDKEKNNKKVPI  183
PlyGRCS_[Staphylococcus_phage_GRCS]                ........................................    0
CF-301_[prophage_of_Streptococcus_suis_89/1591]    ........................................    0
endolysin_[Listeria_phage_vB_LmoS_293]             ...................................MSVLQ    5
PlyAB1_[Acinetobacter_phage_Abp1]                  ........................................    0
Lysostaphin_[Nocardia_seriolae]                    ........................................    0
Consensus

LysP108_[Staphylococcus_phage_P108]                ..........MDKRGK.KPEGMVIHNDAGR.....SSGQQ   24
LysGH15_[Staphylococcus_phage_GH15]                KVSKNHINYTMDKRGK.KPEGMVIHNDAGR.....SSGQQ  224
LysK_[Staphylococcus_phage_K]                      KVSKNHINYTMDKRGK.KPEGMVIHNDAGR.....SSGQQ  224
HydH5_[Staphylococcus_phage_vB_SauS-phiIPLA88]     WKDVKTIKYTISSQVVNYPEYIYHFIVEGNRRLEKPKGIM  223
PlyGRCS_[Staphylococcus_phage_GRCS]                ............MKSQ.QQAKEWIYKHEG.......TGVD   20
CF-301_[prophage_of_Streptococcus_suis_89/1591]    .............MTTVNEALNNVRAQVG.......SGVS   20
endolysin_[Listeria_phage_vB_LmoS_293]             YNYINKNQFSRPGYKLLRVSKIVMHYTAN.......PGAS   38
PlyAB1_[Acinetobacter_phage_Abp1]                  ......................MILTKDG........FSI   10
Lysostaphin_[Nocardia_seriolae]                    ........MSHHRTTANSVTVMDLLGDSGACR....TGTT   28
Consensus

LysP108_[Staphylococcus_phage_P108]                YENSLANAGYARYANGIAHYYGSEGYVWEAIDAKNQIAWH   64
LysGH15_[Staphylococcus_phage_GH15]                YENSLANAGYARYANGIAHYYGSEGYVWEAIDAKNQIAWH  264
LysK_[Staphylococcus_phage_K]                      YENSLANAGYARYANGIAHYYGSEGYVWEAIDAKNQIAWH  264
HydH5_[Staphylococcus_phage_vB_SauS-phiIPLA88]     IRNAQTMSSVENLYNSRKKYKQDVEYPHFYVDRH..NIWA  261
PlyGRCS_[Staphylococcus_phage_GRCS]                FDG..........AYGFQCMDLAVAYVYYITDGK.VRMWG   49
CF-301_[prophage_of_Streptococcus_suis_89/1591]    VGNGE........CYALASWYERMISPDATVGLGAGVGWV   52
endolysin_[Listeria_phage_vB_LmoS_293]             ADN.........HRRYFRDLKERYASAHIFIDDNEAICII   69
PlyAB1_[Acinetobacter_phage_Abp1]                  IRN.........ELFGGKLDQTQVDAINFIVEKATESGLS   41
Lysostaphin_[Nocardia_seriolae]                    SRRRHR..AEPSATDRVKTAAGVAVAAGALIGTATQLVPA   66
Consensus

LysP108_[Staphylococcus_phage_P108]                TGDGTGANSGN.FRFAGIEVCQSMSASDAQFLKNEQAVFQ  103
LysGH15_[Staphylococcus_phage_GH15]                TGDGTGANSGN.FRFAGIEVCQSMSASDAQFLKNEQAVFQ  303
LysK_[Staphylococcus_phage_K]                      TGDGTGANSGN.FRFAGIEVCQSMSASDAQFLKNEQAVFQ  303
HydH5_[Staphylococcus_phage_vB_SauS-phiIPLA88]     PRRAVFEVPNE.PDYIVIDVCEDYSASKNEFIFNEIYAMG  300
PlyGRCS_[Staphylococcus_phage_GRCS]                NAKDAINN.....DFKGLATVYENTPS...FKPQLGDVAV   81
CF-301_[prophage_of_Streptococcus_suis_89/1591]    SGAIGDTIS.....AKNIGSSYNWQANG....WTVSTSGP   83
endolysin_[Listeria_phage_vB_LmoS_293]             PLNEVAYHANE.RSCKLTALQASTSYYRGGNANLTSIGIE  108
PlyAB1_[Acinetobacter_phage_Abp1]                  YPEAAYLLATI.YHETGLPSGYRTMQP....IKEAGSDSY   76
Lysostaphin_[Nocardia_seriolae]                    LAHAAPLLPTHDEKPTAIGEDVAVADTALELKEAKPDAAP  106
Consensus

LysP108_[Staphylococcus_phage_P108]                FTAEKFKEWGLTPNRKTVRLHMEFVPTACPHRSM.VLHTG  142
LysGH15_[Staphylococcus_phage_GH15]                FTAEKFKEWGLTPNRKTVRLHMEFVPTACPHRSM.VLHTG  342
LysK_[Staphylococcus_phage_K]                      FTAEKFKEWGLTPNRKTVRLHMEFVPTACPHRSM.VLHTG  342
HydH5_[Staphylococcus_phage_vB_SauS-phiIPLA88]     VAVDMMVEYEIPLSIENLKVDDSIWRSMLEHVNWNMIDNG  340
PlyGRCS_[Staphylococcus_phage_GRCS]                YTNSQYGHIQCVISG.....NLDYY..TCLEQN..WLGGG  112
CF-301_[prophage_of_Streptococcus_suis_89/1591]    FKAGQIVTLGATPGNP...YGHVVIVEAVDGDRLTILEQN  120
endolysin_[Listeria_phage_vB_LmoS_293]             MCLDKNWNITAATFNRSVDVAAELCKTYDLTPSDIIRHYD  148
PlyAB1_[Acinetobacter_phage_Abp1]                  LRSKKYYPY..........IGYGYVQLTWEENYG..RISK  104
Lysostaphin_[Nocardia_seriolae]                    VAEAAPQAQQVAATPDPAPFGLQNLPPEISAPLQ.QAEQT  145
Consensus
```

Figure S1 (part1)

```
LysP108_[Staphylococcus_phage_P108]                FNPVTQ.GRPSQAIMNKLK..DYFIKQIKNYMDKGTSSST    179
LysGH15_[Staphylococcus_phage_GH15]                FNPVTQ.GRPSQAIMNKLK..DYFIKQIKNYMDKGTSSST    379
LysK_[Staphylococcus_phage_K]                      FNPVTQ.GRPSQAIMNKLK..DYFIKQIKNYMDKGTSSST    379
HydH5_[Staphylococcus_phage_vB_SauS-phiIPLA88]     VPPKDKYEALEKALLNIFKNREKLLNSITKPTVTKSRIKV    380
PlyGRCS_[Staphylococcus_phage_GRCS]                FDGWEK.ATIRTHYYDGVT..HFIRPKFS......ASNSN    143
CF-301_[prophage_of_Streptococcus_suis_89/1591]    YGGKRY...PVRNYYSAASYRQQVVHYIT.......PPGT    150
endolysin_[Listeria_phage_vB_LmoS_293]             VTGKNCPAPWVAKPSELTRFRNAVNAKLKGASQNKNRHDG    188
PlyAB1_[Acinetobacter_phage_Abp1]                  LIGVDLIKNPEKALEPLIAIQIAIKGMLN......GWFTG    138
Lysostaphin_[Nocardia_seriolae]                    LKTLQQQLHPGPNVVKPVAG..QLTSGFG.MRWGAMHYGI    182
Consensus

LysP108_[Staphylococcus_phage_P108]                VVKDGKTSSASTPATRPVTGSWKKNQYGTWYKPENATFVN    219
LysGH15_[Staphylococcus_phage_GH15]                VVKDGKTSSASTPATRPVTGSWKKNQYGTWYKPENATFVN    419
LysK_[Staphylococcus_phage_K]                      VVKDGKTSSASTPATRPVTGSWKKNQYGTWYKPENATFVN    419
HydH5_[Staphylococcus_phage_vB_SauS-phiIPLA88]     MVDNKNADIANVRDSSPTANNGSASKQPQIITETSPYTFK    420
PlyGRCS_[Staphylococcus_phage_GRCS]                VLETSKVN.........TFGNWKQNQYGTYYRNENATFTC    174
CF-301_[prophage_of_Streptococcus_suis_89/1591]    VAQSAPN..........LAGSRSYRETGTMTVTVDALNVR    180
endolysin_[Listeria_phage_vB_LmoS_293]             KVVDSAPLLTKMDFKSSPFRMYKSGTEFLVYEHNQYWYKT    228
PlyAB1_[Acinetobacter_phage_Abp1]                  VGFRRKR..........PVSKYNKQQYVAARNIINGKDKA    168
Lysostaphin_[Nocardia_seriolae]                    DFADPIGTPIHSVMGGTVIDAGPASGFGLWVRVKQDDGTT    222
Consensus

LysP108_[Staphylococcus_phage_P108]                GNQPIVTRIGSPFLNAPVGGN....LPAGATIVYDEVCIQ    255
LysGH15_[Staphylococcus_phage_GH15]                GNQPIVTRIGSPFLNAPVGGN....LPAGATIVYDEVCIQ    455
LysK_[Staphylococcus_phage_K]                      GNQPIVTRIGSPFLNAPVGGN....LPAGATIVYDEVCIQ    455
HydH5_[Staphylococcus_phage_vB_SauS-phiIPLA88]     QALDRQMSRGNPKKSHTWGWANATRAQTSSSMNVKRIWES    460
PlyGRCS_[Staphylococcus_phage_GRCS]                GFLPIFARVGSPKLSEPNGYW....FQPNGYTPYDEVCLS    210
CF-301_[prophage_of_Streptococcus_suis_89/1591]    RAPNTSGEIVAVYKR.............GESFDYDTVIID    207
endolysin_[Listeria_phage_vB_LmoS_293]             YIDDKLYYMYKSFCDVVTKKD.....AKGRIKVRIKSAKD    263
PlyAB1_[Acinetobacter_phage_Abp1]                  ELIAKYAIIFERALRSL.......................    185
Lysostaphin_[Nocardia_seriolae]                    AVYGHVNDMYVSVGQRVNTGD....VIASVGNRGNSTGPH    258
Consensus

LysP108_[Staphylococcus_phage_P108]                A.GHIWIGYNA..YNGNRVYCPVRTCQGVPPN.HIPGVAW    291
LysGH15_[Staphylococcus_phage_GH15]                A.GHIWIGYNA..YNGDRVYCPVRTCQGVPPN.HIPGVAW    491
LysK_[Staphylococcus_phage_K]                      A.GHIWIGYNA..YNGNRVYCPVRTCQGVPPN.QIPGVAW    491
HydH5_[Staphylococcus_phage_vB_SauS-phiIPLA88]     N.TQCYQMLNLGKYQGVSVSSLNKILKGKGTL.NNQGKAF    498
PlyGRCS_[Staphylococcus_phage_GRCS]                D.GLVWIGYN...WQGTRYYLPVRQWNGKTGNSYSIGLPW    246
CF-301_[prophage_of_Streptococcus_suis_89/1591]    VNGYVWVSYIG..GSGKRNYVAT....GATKDGKRFGNAW    241
endolysin_[Listeria_phage_vB_LmoS_293]             LRIPVWNNTKLS..SGKIKWYAPNTKLAWYNNGKGYLELW    301
PlyAB1_[Acinetobacter_phage_Abp1]                  ........................................    185
Lysostaphin_[Nocardia_seriolae]                    LHLEIWDAADN..KMDPLPWLAS........KGVLMQQQW    288
Consensus

LysP108_[Staphylococcus_phage_P108]                GVFK....................................    295
LysGH15_[Staphylococcus_phage_GH15]                GVFK....................................    495
LysK_[Staphylococcus_phage_K]                      GVFK....................................    495
HydH5_[Staphylococcus_phage_vB_SauS-phiIPLA88]     AEACKKHNINEIYLIAHAFLESGYGTSNFASGKDGVYNYF    538
PlyGRCS_[Staphylococcus_phage_GRCS]                GVFS....................................    250
CF-301_[prophage_of_Streptococcus_suis_89/1591]    GTFK....................................    245
endolysin_[Listeria_phage_vB_LmoS_293]             YPSDGWYYTANYFLK.........................    316
PlyAB1_[Acinetobacter_phage_Abp1]                  ........................................    185
Lysostaphin_[Nocardia_seriolae]                    GPDQ....................................    292
Consensus

LysP108_[Staphylococcus_phage_P108]                ........................................    295
LysGH15_[Staphylococcus_phage_GH15]                ........................................    495
LysK_[Staphylococcus_phage_K]                      ........................................    495
HydH5_[Staphylococcus_phage_vB_SauS-phiIPLA88]     GIGAYDNNPNYAMTFARNKGWTSPAKAIMGGASFVRKDYI    578
PlyGRCS_[Staphylococcus_phage_GRCS]                ........................................    250
CF-301_[prophage_of_Streptococcus_suis_89/1591]    ........................................    245
endolysin_[Listeria_phage_vB_LmoS_293]             ........................................    316
PlyAB1_[Acinetobacter_phage_Abp1]                  ........................................    185
Lysostaphin_[Nocardia_seriolae]                    ........................................    292
Consensus

LysP108_[Staphylococcus_phage_P108]                ........................................    295
LysGH15_[Staphylococcus_phage_GH15]                ........................................    495
LysK_[Staphylococcus_phage_K]                      ........................................    495
HydH5_[Staphylococcus_phage_vB_SauS-phiIPLA88]     NKGQNTLYRIRWNPKNPATHQYATAIEWCQHQASTIAKLY    618
PlyGRCS_[Staphylococcus_phage_GRCS]                ........................................    250
CF-301_[prophage_of_Streptococcus_suis_89/1591]    ........................................    245
endolysin_[Listeria_phage_vB_LmoS_293]             ........................................    316
PlyAB1_[Acinetobacter_phage_Abp1]                  ........................................    185
Lysostaphin_[Nocardia_seriolae]                    ........................................    292
Consensus

LysP108_[Staphylococcus_phage_P108]                ...............                             295
LysGH15_[Staphylococcus_phage_GH15]                ...............                             495
LysK_[Staphylococcus_phage_K]                      ...............                             495
HydH5_[Staphylococcus_phage_vB_SauS-phiIPLA88]     KQIGLKGVYFTRDKY                             633
PlyGRCS_[Staphylococcus_phage_GRCS]                ...............                             250
CF-301_[prophage_of_Streptococcus_suis_89/1591]    ...............                             245
endolysin_[Listeria_phage_vB_LmoS_293]             ...............                             316
PlyAB1_[Acinetobacter_phage_Abp1]                  ...............                             185
Lysostaphin_[Nocardia_seriolae]                    ...............                             292
Consensus
```

Figure S1 (part2)

Figure S1. Multiple sequence alignment of LysP108, several endolysins, and lysostaphin.

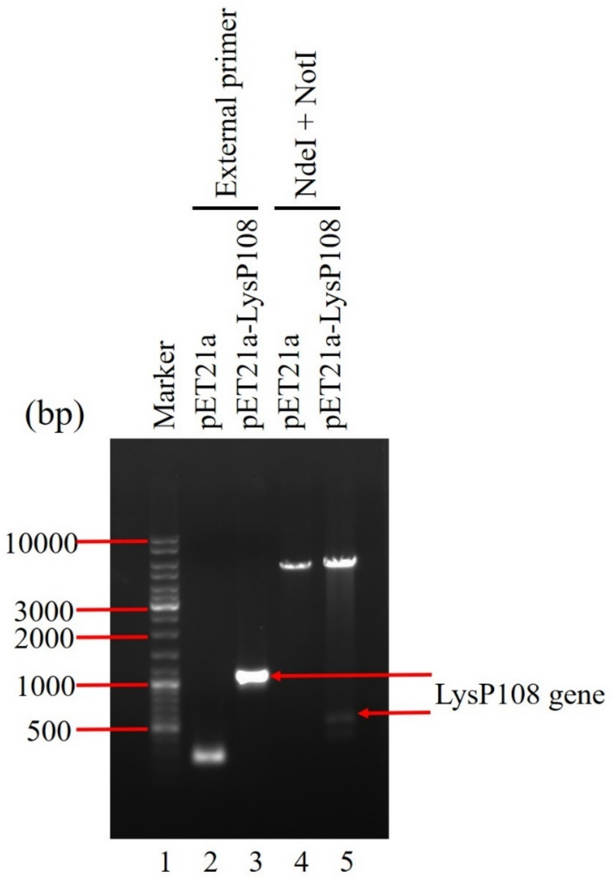

Figure S2. Agarose gel electrophoresis photograph of recombinant plasmid pET21a-LysP108. The plasmid was amplified by external primer, and digested by NdeI and NotI. The red arrows display fragments containing LysP108 gene.

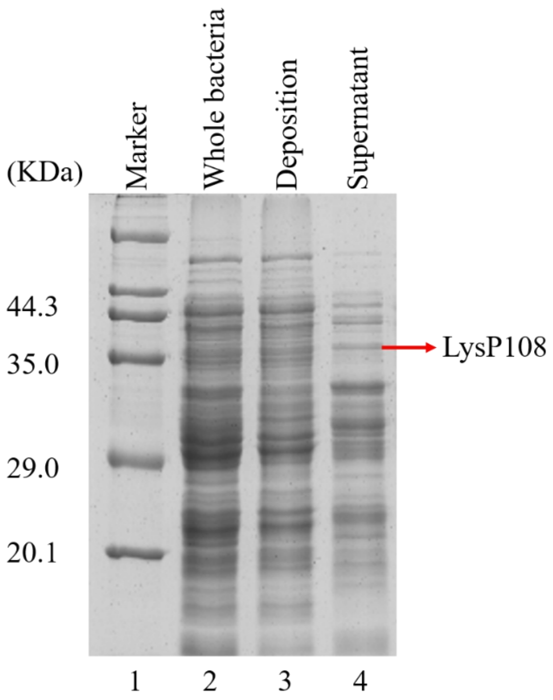

Figure S3. SDS-PAGE photograph of recombinant protein LysP108. The protein was expressed in *E. coli* induced by 0.10 mM IPTG at 37 °C for 5 h. The red arrow indicates the presence of LysP108.

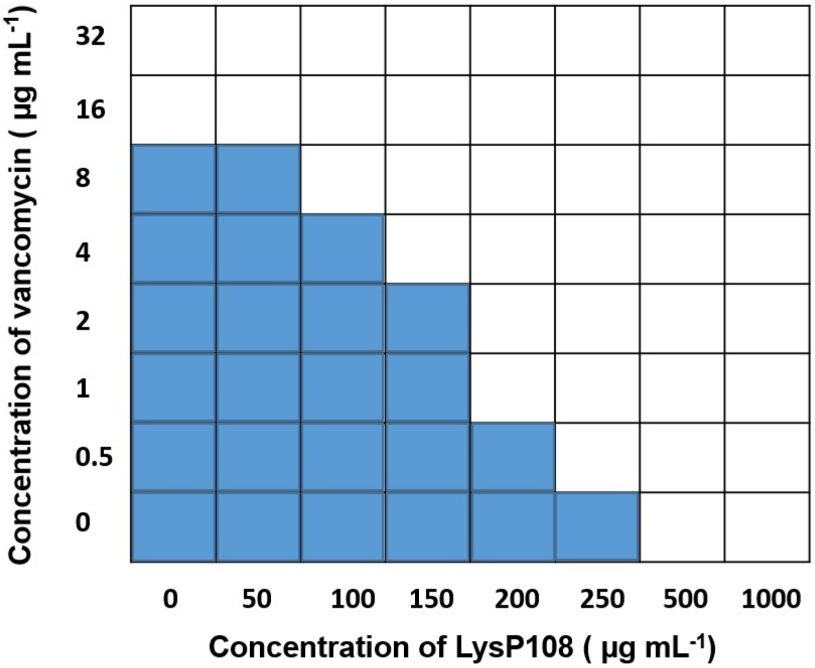

Figure S4. Standard checkerboard broth micro-dilution assay of LysP108 and vancomycin. The blank areas indicated that the growth of MRSA was inhibited by vancomycin and LysP108 at the corresponding concentration, and the blue areas indicated the normal growth of MRSA.
